# Supplementary material for: Unintended exposure to e-liquids and subsequent health outcomes among US youth and adults
Source: PLoS One. 2024 Jul 30;19(7):e0308006. doi: 10.1371/journal.pone.0308006 (PMC11288430; doi:10.1371/journal.pone.0308006)
Supplement: Attachment — Submitted filename: 06.12.24 v3 R3 Letter eliquid exposure.docx [file pone.0308006.s001.docx]

**PONE-D-23-26133R2**: Unintended exposure to e-liquid and subsequent health outcomes among US youth and adults

**Reviewer #3:**

1. This is a brief manuscript that addresses a clear question and provides a strong rationale. It uses publicly available data from a well-established, large-scale, nationally representative survey. More context could be provided in the introduction. Rationale about why these specific age groups were compared would strengthen this paper, as would the use of more high-quality citations in the introduction and discussion.

**Response**: Thank you for your support of this paper. As suggested, we have added a rationale in the Introduction to include the three age strata within the analysis.

We also include reputable citations from sources such as Centers for Disease Control and Prevention and the National Center for Chronic Disease Prevention and Health Promotion (US) Office on Smoking and Health.

See references:

Kramarow EA, Elgaddal N. Current Electronic Cigarette Use Among Adults Aged 18 and Over: United States, 2021. NCHS Data Brief. 2023;(475):1-8.

National Center for Chronic Disease Prevention and Health Promotion (US) Office on Smoking and Health. E-Cigarette Use Among Youth and Young Adults: A Report of the Surgeon General [Internet]. Atlanta (GA): Centers for Disease Control and Prevention (US); 2016. Available from: https://www.ncbi.nlm.nih.gov/books/NBK538680/

**Revised text**:

Page 3, We chose to stratify the analyses by these age groups because previous research has shown that e-cigarette use behaviors (e.g. preferred device type, average frequency of use) vary by age.^1,2^ For example, adolescents (vs. adults) may be more likely to modify e-cigarette devices,^3^ and e-cigarette use is prevalent among young adults (aged 18-24 years).^2^ These behaviors may put adolescents and young adults at risk for greater exposure to e-liquids.

2. The use of consistent terminology (e.g., variable descriptors, device types) throughout would improve readability.

**Response**: We agree with this suggestion and have revised the text accordingly.

3. It would have been nice to see these analyses pre-registered (if they were, please include a reference to the pre-registration in Methods).

**Response**: Unfortunately, this article has not been pre-registered. Plos One does not require pre-registration of secondary data analysis of publicly available national datasets.

4. The methods section is reasonably well structured, although currently missing some key details. Information presented in the methods section does not need to be reiterated in the introduction or results.

**Response**: We revised the Introduction, Methods and Results accordingly to reduce redundancies.

**Revised text**: Please see updated Introduction and Results.

5. To the extent of my knowledge, the analyses appear reasonably rigorous. The results are clearly presented, with three useful tables. Some repetition could be avoided between tables and text (particularly in the first paragraph of the results section).

**Response**: We deleted many redundancies throughout the paragraph (e.g., “who used e-cigarettes in the past 12 months”).

**Revised text**:

Page 6, Among youth (ages 12-17) who used e-cigarettes in the past 12 months, 25.5% reported e-liquid exposure. Of those who reported exposure, 10.3% reported becoming sick, and 3.5% reported going to a hospital. Among young adults (ages 18-24) who used e-cigarettes in the past 12 months, 25.2% reported e-liquid exposure. Of those who reported exposure, 11.0% reported getting sick, and 2.7% reported they went to a hospital. Among older adults (ages ≥25) who used e-cigarettes in the past 12 months, 19.1% reported e-liquid exposure. Of those who reported exposure, 14.0% reported getting sick, and 6.8% reported that they went to a hospital (**Table 1**).

6. Some seemingly important findings are not discussed. Frequency of use appears to be an important variable, but other than saying it was a covariate, I do not see it mentioned in the results of the discussion. Similarly, going to hospital rates are twice as high among older adults than the other age groups, but this is not discussed.

**Response**: Thank you for your suggestions. We added discussion about these findings.

**Revised text**:

Page 8, We observed age-related differences prevalence rates ofe-liquid exposures. For example, youth and young adults had a higher prevalence of e-liquid exposure than older adults. Further, higher e-cigarette use frequency was related to higher odds of e-liquid exposure, but this association was not observed in older age groups. Although increased e-cigarette use frequency may be related to frequent refills of e-liquids in both younger and older individauls, it is possible that younger individuals are less experienced than older adults in safely handling e-cigarette devices and liquids, . It is worth noting that although older adults had the lowest prevalence of e-liquid exposure tthey reported the highest prevalence of “becoming sick” and “going to a hospital” after the exposure This may suggest that the severity of e-liquid exposure might be worse among older adults than younger people, necessitating a greater level of medical attention. Alternatively, youth may experience similar types of e-liquid exposure, but be less likely to present to the hostipal due to confidentiality concerns to the hospitals and may hide their e-liquid exposure from parents and may go to the hospital for greater symptom severity. Future studies should use clinical data (e.g., electronic health records) to identify specific symptoms and severity of symptoms related to e-liquid exposure.

Page 8, Our study also highlights sociodemographic disparities in e-liquid exposures. Among young adults, Non-Hispanic individuals and those with higher income levels had higher odds of e-liquid exposure to e-liquid. In general, more frequent use of e-cigarettes may be associated with increased risks of e-liquid exposure. Existing data indicate that e-cigarettes are more likely to be used by non-Hispanic White individuals, and those from higher income levels among young adults.^2^ Thus, certain education and communication related to harmful effects and risks of e-liquid exposure could be targeted to populations who are at greater risk for using e-cigarettes.

7. In the abstract, I suggest including clear and specific definitions of groups and variables in the abstract, e.g., specifying the age range of ‘youth’, ‘young adults’, and ‘older adults’, defining ‘becoming sick’.

**Response:** We defined the age groups. “Becoming sick” was not defined in the PATH Study. Thus, we have added this to the limitation.

**Revised text:**

Page 2, We also examined associations between these outcomes and the device type used (refillable tank /mod system, replaceable prefilled cartridges, disposable/ other device type). E-liquid exposure was reported by 25% of youth (aged 12-17 years), 25% of young adults (aged 18-24 years), and 19% of older adults (aged≥ 25 years).

Page 9, Survey questions did not assess the specific route of exposure, the severity of the exposure or subsequent sickness, or if going to the hospital resulted in hospital admission or other medical interventions. Future studies should examine these factors since the health impact and harm could vary by route and extent of exposure.

8. The phrase “vs a disposable device and something else” needs to be clarified; I understand this is the reference group including two collapsed options but the current phrasing is confusing without context from methods. For instance, it could be phrased as: “vs people who used other device types including disposables” and combined in the same parentheses as the aOR/CI.

**Response:** We revised the sentence.

**Revised text:**

Page 2, Among young adults, the use of a refillable tank /mod system was associated with higher odds of e-liquid exposure (aOR=2.2, 95% CI=1.2, 4.1) than the use of other device types, including disposables.

9. In the abstract the conclusion is a little narrow (warning labels only); consider other packaging regulations too, and even device/bottle designs which could avoid leaks (this can be explored in more details in the discussion, with the caveat that you don't know the exact cause of exposure).

**Response:** We revised the abstract accordingly.

**Revised text:**

Page 2, The findings suggest that, at a minimum, e-cigarettes/e-liquids may need warning labels that state the risks of e-liquid exposure and packaging regulations that promote device and bottle designs that minimize e-liquid spills.

10. Introduction: I cannot find the number of cases cited in the linked source. It would also help to specify the timeframe this number refers to (e.g., 3,864 new cases over the past year?)

**Response:** We updated the number with most recent data.

**Revised text:**

Page 3, Between 2011 and 2024, 50,743 e-cigarette- and nicotine liquid-related exposure cases were reported to Poison Control Centers in the United States.^4^

11. I suggest not specifying past 12 months/ past 30 days in this paragraph, because the discrepancy between to two raises questions which are not answered until the methods section, and it is redundant if specified below.

**Response:** We deleted this part.

12. Last sentence: Clearly state the comparators here; ‘tank and mod devices allow individuals to easily refill the device’ as compared to which devices? Disposables, all other devices?

**Response:** We deleted this sentence in the revision.

13. Providing some background on the types of devices available and their characteristics may strengthen the rationale for this hypothesis while also providing clarifications for those less familiar with the e-cig market.

**Response:** We added the rationale for the study.

**Revised text:**

Page 3, We also examined associations between the outcomes and the device type used (refillable tank /mod system, replaceable prefilled cartridges, disposable/other device type) to explore whether the ability to manually manipulate e-liquids through certain product features might affect the risk of exposure. Understanding e-liquid exposure and related outcomes by device types is important since individuals can access a variety of e-cigarette device types in the United States and e-cigarette popularity has been changed in recent years such that disposable and mod systems are frequently used by adolescents and young adults in recent years.^5^ Further, mod systems might put people at greater risk for e-liquid exposure since people are directly putting e-liquids in their devices. Although closed devices such as disposables/cartridges have lower likelihood of e-liquid exposure since e-liquid is prefilled, research shows that youth and young adults are manipulating or ‘hacking’ these devices to put their own e-liquids, which may increase their e-liquid exposure.^3^

14. In the introduction it states “We hypothesized that using refillable tank or mod system e-cigarette devices would be associated with a higher likelihood of e-liquid exposures, given that these devices allow individuals to easily refill the device with e-liquids”, then in the discussion “Consistent with our hypothesis, use of refillable tank/mod systems or replaceable prefilled cartridges (vs. disposable devices or something else) was associated with higher odds of e-liquid exposure among young adults”.

I don’t see how prefilled cartridge models are ‘consistent’ in this case; they were not mentioned in the hypothesis, and they do not require directly handling e-liquids.

**Response:** We revised this sentence.

**Revised text:**

Page 8, We also found that the use of refillable tank/mod systems or replaceable prefilled cartridges (vs. disposable / other device type) was associated with higher odds of e-liquid exposure among young adults.

15. Paragraph 1: Please provide additional details about PATH methodology or add a reference to a paper which does. All data used in this study was self-reported via surveys this should be specified in the opening paragraph.

**Response:** We added more details about the PATH Study.

**Revised text:**

Page 4, We conducted a secondary data analysis of the US nationally representative Population Assessment of Tobacco and Health (PATH) Study Wave 5 (2018-2019) youth (aged 12-17 years) and adult (aged 18 years and older) datasets. The PATH Study public dataset’s self-report measures are well-validated, and the details of the study methodology, eligibility criteria, measures, and theoretical framework are described elsewhere.^6,7^ The PATH uses multi-stage sampling to allow the calculation of US population estimates.

16. Please specify which statistical software/language was used.

**Response:** We specified the statistical program was used.

**Revised text:**

Page 6, We used STATA 18.0 (College Station, TX) for statistical analyses.

17. ‘becoming sick with devices with…’: rephrase e.g., ‘Older adults who used devices with refillable tanks, mod systems or replaceable prefilled cartridges had lower odds of becoming sick’

**Response:** We revised this sentence.

**Revised text:**

Page 7, Among older adults who were exposed to e-liquid, use of refillable tank/mod systems (aOR=0.1, 95% CI=0.0, 0.5) or replaceable prefilled cartridge devices (aOR=0.1, 95% CI=0.0, 0.5) (vs. disposable/other device type) was associated with lower odds of becoming sick after an e-liquid exposure.

18. The limitations should mention how broad ‘becoming sick’ is as a measure

**Response:** We already had this limitation but elaborate them in the revision.

**Revised text:**

Page 9, PATH survey data may be prone to self-report, recall, or social desirability bias. Survey questions did not assess the specific route of exposure, the severity of the exposure or subsequent sickness, or if going to the hospital resulted in hospital admission or other medical interventions.

19. “Did not assess .. whether it required hospital admission” – slightly confusing phrasing since it did assess if people went to hospital. Perhaps rephrase to clarify it didn’t assess if these hospitalizations were necessary?

**Response:** We revised the sentence.

**Revised text:**

Page 9, Survey questions did not assess the specific route of exposure, the severity of the exposure or subsequent sickness, or if going to the hospital resulted in hospital admission or other medical interventions. Future studies should examine these factors since the health impact and harm could vary by route and extent of exposure.

20. Could cite <https://pubmed.ncbi.nlm.nih.gov/35622007/>

**Response:** We cited this paper in the introduction.

**Revised text:**

Page 3, We also examined associations between the outcomes and the device type used (refillable tank /mod system, replaceable prefilled cartridges, disposable/other device type) to explore whether the ability to manually manipulate e-liquids through certain product features might affect the risk of exposure. Understanding e-liquid exposure and related outcomes by device types is important since individuals can access a variety of e-cigarette device types in the United States and e-cigarette popularity has been changed in recent years such that disposable and mod systems are frequently used by adolescents and young adults in recent years.^5^ Further, mod systems might put people at greater risk for e-liquid exposure since people are directly putting e-liquids in their devices. Although closed devices such as disposables/cartridges have lower likelihood of e-liquid exposure since e-liquid is prefilled, research shows that youth and young adults are manipulating or ‘hacking’ these devices to put their own e-liquids, which may increase their e-liquid exposure.^3^

21. “especially directed at specific device types”: would be good to elaborate on this point.

**Response:** We revised the sentence.

**Revised text:**

Page 9, This study provides one of the first estimates of e-liquid exposure and potential health burdens from the exposure among youth and adults using US nationally representative samples. Overall, the findings highlight the need for effective e-liquid packaging and labeling regulations. This could include warning labels to inform potential risk of e-liquid exposure, e-cigarette device, and e-liquid bottle design to prevent accidental exposure to e-liquids. Our findings also suggest that e-liquid injury prevention strategies may need to be directed toward refillable tank/mod systems and individuals who frequently use e-cigarettes. Moreoever, future research is needed to better characterize the clinical severity of e-liquid exposures and how they occur (e.g., while refilling or mixing e-liquids).

22. Reference (9) needs context, not entirely clear why cited here.

**Response:** We removed this citation.

23. “further research on how these exposures occur”: this is vague, I would opt for more specific phrasing, particularly as a closing statement

**Response:** We revised the sentence.

**Revised text:**

Page 8, We observed age-related differences prevalence rates ofe-liquid exposures. For example, youth and young adults had a higher prevalence of e-liquid exposure than older adults. Further, higher e-cigarette use frequency was related to higher odds of e-liquid exposure, but this association was not observed in older age groups. Although increased e-cigarette use frequency may be related to frequent refills of e-liquids in both younger and older individauls, it is possible that younger individuals are less experienced than older adults in safely handling e-cigarette devices and liquids, . It is worth noting that although older adults had the lowest prevalence of e-liquid exposure tthey reported the highest prevalence of “becoming sick” and “going to a hospital” after the exposure This may suggest that the severity of e-liquid exposure might be worse among older adults than younger people, necessitating a greater level of medical attention. Alternatively, youth may experience similar types of e-liquid exposure, but be less likely to present to the hostipal due to confidentiality concerns to the hospitals and may hide their e-liquid exposure from parents and may go to the hospital for greater symptom severity. Future studies should use clinical data (e.g., electronic health records) to identify specific symptoms and severity of symptoms related to e-liquid exposure.

**24. We also have made the following grammatical and typographical revisions, as suggested by Reviewer 3.**

- Removed ‘however’ on the 9th line.
- Rephrased ‘children less than age 5’ to (‘aged 5 and below’, ‘under the age of 5’…)
- Updated second to last sentence: ‘influence of device type used and exposure to e-liquids and on subsequent outcomes’.
- Paragraph 2: Removed extra semi-colon before ‘in comparison’
- Added the word ‘question’ to: “We selected past-30-day e-cigarette users as an analytic sample in these regression models since the most often used device type question was only asked to past-30-day e-cigarette users”
- Updated the wording of the following sentence to: “For youth sample, education and income covariates refer to parental education and parental income, and the insurance status was not recorded.
- Added ‘covariates’ and ‘outcomes’ in Table 2’s title
- Paragraph 1: Corrected 7th line typo “e-liqiud”
- **Updated** inconsistent variable descriptions: “(vs. disposable devices/ something else)” and “(vs. disposable/other devices)”

**Reviewer #4:**

This analysis will be a valuable contribution to the literature; a few small suggestions are provided below.

1. Abstract. The final sentence suggests that warning labels informing users of the risk of e-cigarette liquid exposure are needed. While perhaps true, this would seem to be the minimum needed to address the problem, which your data show is encountered by 20-25% of past-year users. Consider saying, "The findings suggest that, at a minimum, e-cigarettes/e-liquids..." Alternatively, consider a broader sentence similar to that which concludes the manuscript: "The findings also highlight the need for consistent and strong enforcement of e-liquid packaging and labeling regulations..."

**Response:**

Thank you for this helpful suggestion. We have updated the Abstract as suggested.

**Revised text:**

Page 2, The findings suggest that, at a minimum, e-cigarettes/e-liquids may need warning labels that state the risks of e-liquid exposure and packaging regulations that promote device and bottle designs that minimize e-liquid spills.

2. Page 6, Re: "up to 25% of individuals...report oral, ocular, and dermal e-liquid exposure." Based on the PATH question, the "and" should be "or."

**Response:** We revised the sentence per request.

3. Page 6/7 Re: the phrase, ".... and few reported going to the hospital." I realize the numbers are small, but the phrasing understates your finding. Suggest either, "...a few reported..." OR "a small percentage of these reported..."

**Response:** We revised the sentence per request.

4. Finally, I would reiterate in the Discussion section that these findings are from a US based nationally representative sample. Given the broad reach of use of e-cigarettes, data from other countries will be useful.

**Response:** We added this to the limitation.

**Revised text:**

Page 10, Given the broad reach of the use of e-cigarettes, future research is warranted with data from other countries.

**Reviewer #5:**

This study provides valuable insights into the prevalence and potential consequences of e-liquid exposure. The following are my suggestions for the authors.

1. The methods section lacks sufficient detail. Inclusion and exclusion criteria for the study population should be clearly outlined.

**Response:** We added these details.

**Revised text:** Please see updated “Measures” and “Analytical strategy”

2. Additionally, there is a lack of specificity regarding e-liquid exposure, including assessing the severity or extent of exposure, which could impact the resulting health consequences.

**Response:** Based on the reviewers’ comments, we further elaborate this limitation. Unfortunately, the PATH Study does not have specific questions regarding severity or extent of e-liquid exposure. Even without the severity or extent of exposure variables, this study is still important because it provides information to clinicians about the extent to which people report being sick or going to the hospital from e-liquid exposure. They can also inform tobacco regulatory policies regarding e-liquid exposure such as warning labels to inform potential risk of e-liquid exposure, e-cigarette device, and e-liquid bottle design to prevent accidental exposure to e-liquids. Future studies should use clinical data (e.g., electronic health records) to characterize the severity or extent of e-liquid exposure. We added this as a future research direction.

**Revised text:**

Page 9, Survey questions did not assess the specific route of exposure, the severity of the exposure, or if going to the hospital resulted in hospital admission or other medical interventions. Future studies should examine these factors since the health impact and harm could vary by route and extent of exposure.

Page 8, Future studies should use clinical data (e.g., electronic health records) to identify specific symptoms and severity of symptoms related to e-liquid exposure.

3. The study heavily relies on self-reported data from survey respondents, which introduces the possibility of recall bias or social desirability bias. This may lead to inaccurate reporting of e-liquid exposure and related outcomes, thereby affecting the reliability of the findings.

**Response:** Based on the reviewers’ comments, we further elaborate this limitation. Despite the potential for self-report bias, this study is still important because it provides information to clinicians about the extent to which people report being sick or going to the hospital from e-liquid exposure. This study can also inform tobacco regulatory policies regarding e-liquid exposure such as warning labels to inform potential risk of e-liquid exposure, e-cigarette device, and e-liquid bottle design to prevent accidental exposure to e-liquids. Future studies should use clinical data (e.g., electronic health records) to characterize the severity or extent of e-liquid exposure. We added this as a future research direction.

**Revised text**:

Page 9, PATH survey data may be prone to self-report, recall, or social desirability bias. Survey questions did not assess the specific route of exposure, the severity of the exposure or subsequent sickness, or if going to the hospital resulted in hospital admission or other medical interventions. Future studies should examine these factors since the health impact and harm could vary by route and extent of exposure.

Page 8, Future studies should use clinical data (e.g., electronic health records) to identify specific symptoms and severity of symptoms related to e-liquid exposure.

4. Potential confounding factors, such as nicotine dependence, risk-taking behaviors, or specific product characteristics, should have been addressed. Failure to account for these factors could compromise the validity of the study's conclusions regarding the prevalence and consequences of e-liquid exposure.

**Response:**

Thank you for your suggestions. Even though those variables might be related to our study outcomes, we were unable to include or decided not to include those variables.

- The “nicotine dependence” variable is collinear with the “frequency of e-cigarette use in the past 30 days” which was already included in our current model.
- The “risk taking behaviors” were not assessed in PATH Study Wave 5.
- Regarding specific product characteristics, we already included and focused on device type in our study.

Despite this limitation, this study provides one of the first estimates of the population health burden of e-liquid exposures among youth and adults using US nationally representative samples. The findings of this study is still important and valuable to inform future clinical implications and tobacco regulatory policies related to preventing e-liquid exposure and related injuries.

5. The mention of "Consistent with our hypothesis..." in line 121 implies the existence of a study hypothesis, which should have been clearly stated in the Introduction section for transparency and clarity.

**Response:** We removed this statement.

6. Table 1 reports income data for youth aged 12-17, which raises questions about the validity of the survey instrument. Additionally, the mention of income for this age group seems out of context. If included, it should be clarified whether this refers to personal income or household income, and if it is indeed yearly income.

**Response:** We clarified this in the revision**.** In the youth sample, education and income variables refer to parental education and household annual income, which was reported by their parents. Including parental income is still important since parents’ socioeconomic status is related to youth e-cigarette use.

Reference: Sunday, S., Clancy, L. & Hanafin, J. The associations of parental smoking, quitting and habitus with teenager e-cigarette, smoking, alcohol and other drug use in GUI Cohort ’98. Sci Rep 13, 20105 (2023). https://doi.org/10.1038/s41598-023-47061-4

**Revised text:**

Page 6, In the youth sample, education and income covariates refer to parental education and household annual income (reported by parents), and the insurance status was not recorded (Please see **Table 1**).

7. The implications of the study findings for tobacco control efforts should be thoroughly discussed. Specifically, how the results can contribute to better understanding and addressing e-liquid exposure among different age groups, and how this understanding can inform more effective tobacco control strategies.

**Response:** We revised the conclusions and implications of the study.

**Revised text:**

Page 9, This study provides one of the first estimates of e-liquid exposure and potential health burdens from the exposure among youth and adults using US nationally representative samples. Overall, the findings highlight the need for consistent e-liquid packaging and labeling regulations, such as those related to warning labels, e-cigarette device design, and e-liquid bottle design. Our findings also suggest that e-liquid injury prevention strategies may need to be directed toward refillable tank/mod systems and individuals who frequently use e-cigarettes. Moreoever, future research is needed to better characterize the clinical severity of e-liquid exposures and how they occur (e.g., while refilling or mixing e-liquids). Particularly, examinations of symptoms among older adults are warranted to inform clinical practice regarding e-cigarette-related injuries.
